# Pilot randomized controlled trial of acetylsalicylic acid to reduce cerebral microembolism in Chagas heart failure

**DOI:** 10.1055/s-0045-1812028

**Published:** 2025-10-27

**Authors:** Renan Carvalho Castello-Branco, Cárita Victoria Carvalho de Santana, Victor L. P. P. Botelho, Paulo R. S. P. deSousa, Maria C.P. Nunes, Karen L. Furie, Jamary Oliveira-Filho

**Affiliations:** 1 Universidade Federal da Bahia, Hospital Universitario Professor Edgard Santos, Pós-Graduação em Ciências da Saúde, Salvador BA, Brazil.; 2 Universidade Federal de Minas Gerais, Faculdade de Medicina, Ambulatório de Insuficiência Cardíaca, Belo Horizonte MG, Brazil.; 3 Brown University, Neurology Service, Providence RI, United States.

**Keywords:** Cardiomyopathies, Chagas Disease, Cerebrovascular Disorders

## Abstract

**Background:**

Chagas disease is an important cause of heart failure (HF) and stroke, affecting over 6 million people. High-intensity transient signals (HITS) are detected on transcranial Doppler (TCD) in patients with Chagas disease, but the effect of antithrombotic treatment on HITS is unknown.

**Objective:**

To evaluate whether acetylsalicylic acid (ASA) reduces the frequency and number of HITS in patients with Chagasic HF.

**Methods:**

Proof-of-principle pilot prospective, randomized, open, blinded endpoint (PROBE) clinical trial, in which patients with both Chagas and HITS were randomized 2:1 to ASA 300 mg for 7 days and standard HF treatment or standard HF treatment alone (control group). The primary outcome was the proportion of HITS after one week, analyzed using the Chi-squared test.

**Results:**

A total of 373 patients with HF were evaluated, with HITS occurring in 22/190 (12%) Chagasic patients and in 16/183 (8%) non-Chagasic patients ( *p* = 0.531). Twelve of the 22 (54%) Chagasic patients were randomized to treatment with (n = 8) or without ASA (n = 4). Two patients in the control group (50%) persisted with HITS after 7 days of treatment, compared to none in the ASA group, *p* = 0.028. The median number of HITS decreased from 3.5 to 0 with ASA ( *p* = 0.012) and 4.0 to 0.5 in the control group ( *p* = 0.095), with no significant between-group difference ( *p* = 0.262). No adverse events were reported.

**Conclusion:**

In the present pilot clinical trial, ASA reduced the proportion of HITS in patients with Chagas disease HF.

## INTRODUCTION

Chagas disease affects over 6 million people worldwide, mainly in Latin American countries; however, migration patterns have increased the number of infected individuals in Europe, Japan, Australia, Canada, and the United States. 1 Victims are often infected as children, and up to a third eventually develop cardiomyopathy for the rest of their lives, increasing the risk of sudden death from arrhythmias and cerebral infarction. 2 3

The establishment of non-invasive methods to stratify the risk of stroke in patients with Chagas disease is a priority. Additionally, it is important to define alternative outcomes to test new treatments for stroke prevention in this population. Transcranial Doppler (TCD) is a non-invasive and safe method that allows the identification of microembolism in the cerebral circulation in real time. Inflammation and secondary activation of the hemostatic system is thought to increase the risk of thrombus formation and subsequent embolic stroke, especially in the presence of structural myocardial damage such as dilated cardiomyopathy. 4 5 The microembolic events are also called high-intensity transient signals (HITS), visualized on TCD as high-intensity signals (usually > 3 dB above its basal value), unidirectional, transient and with a specific sound, 6 with HITS as a surrogate marker for stroke risk. 7 8 9

In patients with heart failure (HF), HITS occurs more frequently in Chagas when compared to non-Chagas patients, 10 but it is unknown whether this increased risk can be modified by anti-thrombotic medications. In this sense, the aim of the study was to test the effectiveness of acetylsalicylic acid (ASA) in reducing the rate of HITS in patients with Chagas-associated HF.

## METHODS

### Study design and patients

This was a pilot proof-of-principle prospective, randomized, open-label, blinded clinical (PROBE) trial. The patients were assigned a 4-digit alphanumeric identification number consisting of a number (001–999) followed by the first letter of the patient's last name (for data quality monitoring). The system allowed monitoring of the accuracy of sample labeling, preserving confidentiality. The inclusion criteria were age above 18 years of age with HF defined according to Framingham clinical criteria formed by signs (progressive edema of the lower limbs and hepatomegaly not attributable to other diseases) and symptoms (dyspnea on exertion and paroxysmal nocturnal dyspnea) and classified according to the New York Heart Association functional class. 11 12 In addition, patients with Chagas disease had two positive serological tests (enzyme-linked immunosorbent assay [ELISA], immunofluorescence, or hemagglutination assays). The patients were enrolled from the Professor Francisco Magalhães Neto Outpatient Clinic (Hospital Universitario Professor Edgard Santos - HUPES complex) and HF outpatient clinic at Hospital Ana Nery, Salvador, Bahia.

Patients with a history of stroke or atrial fibrillation related to cardiomyopathy were excluded.

The exclusion criteria were designed to avoid confounding variables for TCD HITS detection, such as comorbidities and use of oral anticoagulants. Patients with a history of untreated malignant neoplasm (except localized neoplasm of the skin), ischemic cerebrovascular disease (determined using the Questionnaire for Verifying Stroke-Free Status), 13 chronic dialysis renal failure or end-stage liver failure were excluded. Patients with HITS detected in the TCD were screened for exclusion criteria for using ASA.

Patients screened following the inclusion/exclusion criteria underwent TCD monitoring with a helmet on a Nicolet Companion III TCD system (Natus Medical Inc.). A single investigator performed all tests, blinded to all clinical data. The middle cerebral artery (MCA) was monitored continuously unilaterally for one hour looking for the occurrence of HITS. The proximal MCA was insonated at depth of insonation of 50 to 55 mm using an embolus detection software. All events flagged as HITS were reviewed by the same investigator and were only recorded as HITS when they fulfilled the criteria of a signal at least 9 dB higher in intensity than the surrounding blood, were unidirectional and associated with a characteristic chirping sound on the audio output. Chagasic patients with HITS detected during monitoring were randomly allocated 2:1 to treatment with ASA 300 mg for 7 days and their usual medications for HF treatment or their medications for HF treatment alone. These patients performed a second TCD after seven days to verify the effectiveness of ASA in reducing HITS with an investigator blinded to treatment allocation. The TCD assessor was blinded throughout the process of patient selection, scheduling of exams, randomization, 7-day follow-up, provision of medication, rescheduling of the second exam. In addition, throughout the exam process, the etiology, medications being used and regularity of medications were not questioned by the TCD assessor. Acetylsalicylic acid is a drug widely used in primary and secondary prevention for most cardiovascular and cerebrovascular diseases, with a known safety profile and cost compared to those of other antiplatelet agents. The study favored ASA due to its antithrombotic and antiinflammatory effects, based on the pathophysiological characteristics of patients with Chagas disease with the profile defined in the inclusion criteria (for example: patients with left ventricular thrombus who already have evidence of benefit from anticoagulation or who are already effectively anticoagulated were excluded from the study), with a low risk of bleeding events. Antiplatelet therapy was chosen in view of the prothrombotic profile and higher bleeding rate of patients with Chagas disease associated with the greater intrinsic risk of bleeding from anticoagulation in relation to antiplatelet therapy. 7 8 9

The duration of seven days of treatment was based on the expected efficacy of platelet inhibition in this short interval, which should reduce the dropout rates to a minimum number.

### Statistical analysis

The clinical data collected in Brazil was entered into a Research Electronic Data Capture (REDCap) database and analyzed using STATA version 14.1 (StataCorp). Demographic and clinical characteristics were described as absolute and relative frequencies for categorical variables (and mean/standard deviation or median/interquartile range for continuous variables, depending on its distribution). The normality of continuous variables was defined based on the Kolmogorov-Smirnov test. Based on our preliminary data on the frequency of HITS in cases and controls (14% and 2%, respectively), we expected to recruit 37 Chagasic patients with HITS. Considering a 15% refusal rate, we expected to randomize 30 patients for ASA or control treatment. With this sample size, we expected to have 80% power to detect a minimum reduction of 58% in the proportion of HITS in the ASA group compared to 10% in the control group. Unfortunately, the study was interrupted prematurely due to lack of funding and higher-than-expected refusal rates.

To compare the proportion of HITS between groups, we used the Chi-squared test with Yates correction. As a secondary analysis, we compared the median number of emboli using the Mann-Whitney U test. To compare the proportion of HITS between Chagas and non-Chagas groups, we used logistic regression analysis to control for confounding factors.

### Standard protocol approvals, registrations, and patient consent

The study was approved on March 25, 2011, by the Ethics Committee of HUPES - Universidade Federal da Bahia, Brazil, with the number 240/2011, and all patients signed the informed consent form. The study was conducted in accordance with the Declaration of Helsinki. This clinical trial and its analysis plan were registered at www.clinicaltrials.gov under number NCT01650792.

## RESULTS

Of the patients included in the study, 373 underwent TCD, 190 (51%) with HF Chagas disease, median age of 59 (interquartile range 35–65), 313 (83.9%) afrodescendants, and 123 (65%) females. The most frequent cerebrovascular risk factor was hypertension, present in 116 (62%) patients. Median left ventricular ejection fraction was 42% (interquartile range 28–62). Chagas patients less frequently had comorbidities associated with other HF etiologies, such as coronary artery disease, hypertension, and diabetes. Table 1 shows the baseline characteristics of both populations.

**Table 1:** Baseline characteristics of patients with heart failure of Chagasic versus non-Chagasic etiology

		Total (n = 373)	Chagas disease heart failure (n = 190)	Non-Chagas heart failure (n = 183)	*P* -value
Age in years, median (IQR)		59 (35–65)	55 (50–61)	54 (43–63)	0.094
Female, n (%)		208 (56)	123(65)	85(46)	< 0.001
Race	Asian	6 (1.6)	5 (2.6)	1 (0.5)	< 0.001
	White	39 (10.4)	15 (7.9)	24 (13.1)	
	Afrodescendants	313 (83.9)	135 (71.0)	178 (97.2)	
Hypertension, n (%)		243 (65)	116 (62)	121(68)	< 0.001
Diabetes mellitus, n (%)		75(20)	33 (17)	41(23)	< 0.001
Smoking, n (%)		123 (31)	63 (33.0)	60 (33)	0.308
Use of antiplatelet agents, n (%)		87 (23)	34 (18)	49 (27)	< 0.001
Use of statins, n (%)		91 (24)	48 (25)	40 (22)	< 0.001
Coronary artery disease, n (%)		73 (20)	24 (13)	47 (26)	< 0.001
Functional class, n (%)	I	101 (29)	54 (30)	45 (28)	< 0.001
	II	153 (44)	81 (46)	63 (39)	
	III	67 (19)	31 (18)	36 (22)	
	IV	25 (7)	10 (5)	13 (8)	
Left ventricle ejection fraction in % - median (IQR)		42 (28–62)	50 (37–67)	37 (29–47)	0.919
Patients with high-intensity transient signals, n (%)		41 (11%)	22 (12%)	16 (8.0%)	0.531

Abbreviation: IQR: interquartile range.

Note: The percentages may be unstable due to the small sample size.

Of the patients who underwent TCD, 41 (11%) patients presented with HITS, observed in 22/190 (12%) of Chagasic patients and in 16/183 (8.0%) of non-Chagasic patients ( *p* = 0.531). In the multivariable analysis adjusted for age, sex, and left ventricle ejection fraction, Chagas disease was not associated with HITS (odds ratio = 1.33; 95%CI = 0.60–2.95).

**Figure 1:**
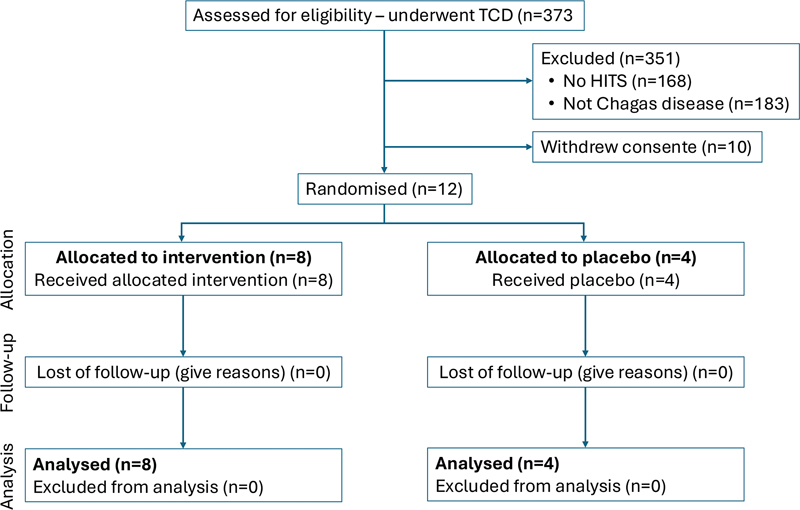
Consolidated Standards of Reporting Trials flowchart.

Twelve of the 22 patients (54.5%) with Chagas disease were randomized to treatment with ASA (n = 8) or a control group (n = 4) ( Figure 1 ). The characteristics of both groups were similar ( Table 2 ). The remaining patients refused consent mostly due to logistical issues (e.g., lived in a city far from the stroke center). After 7 days, 2 patients in the control group (50%) persisted with HITS versus none in the ASA group, *p* = 0.028. No adverse events were reported.

**Table 2:** Primary outcome and clinical characteristics of Chagas disease patients randomized to acetylsalicylic acid (ASA) or control groups

		Total (n = 12)	ASA (n = 8)	Control group (n = 4)	*P* -value
Primary outcome: Patients with HITS at 7 days, n (%)		2 (16.7)	0 (0)	2 (50)	0.028
Age in years, median (IQR)		60 (55–70)	61 (57–73)	59 (52–70)	0.213
Female, n (%)		11 (92)	7 (87)	4 (100)	0.460
Race	Asian	0 (0)	0 (0)	(0)	0.221
	White	0 (0)	0 (0)	(0)	
	Afrodescendants	12 (100)	8 (100)	4 (100)	
Hypertension, n (%)		10 (83)	7 (87)	3 (75)	0.584
Diabetes mellitus, n (%)		2 (17)	1 (12)	1 (25)	0.584
Smoking, n (%)	Smokes	0	0	0	0.679
	Smoked	7 (58)	5 (62)	2 (50)	
	Non-smoking	5 (42)	3 (37)	2 (50)	
Use of antiplatelet agents, n (%)		3 (25)	2 (25)	1 (25)	0.764
Use of statins, n (%)		4 (33.3)	2 (25)	2 (50)	0.386
Coronary artery disease, n (%)		2 (16)	2 (25)	0 (0)	0.273
Functional class, n (%)	I	1 (11)	1 (14)	0 (0)	0.687
	II	5 (55)	4 (57)	1 (50)	
	III	2 (22)	1 (14)	1 (50)	
	IV	1 (11)	1 (14)	0 (0)	
Left ventricle ejection fraction in % - median (IQR)		67 (60.7–70)	66.9 (60.7–70)	67.6(61.6–71.8)	0.358

Abbreviations: HITS, high-intensity transient signals; IQR, interquartile range.

Note: The percentages may be unstable due to the small sample size.

**Figure 2:**
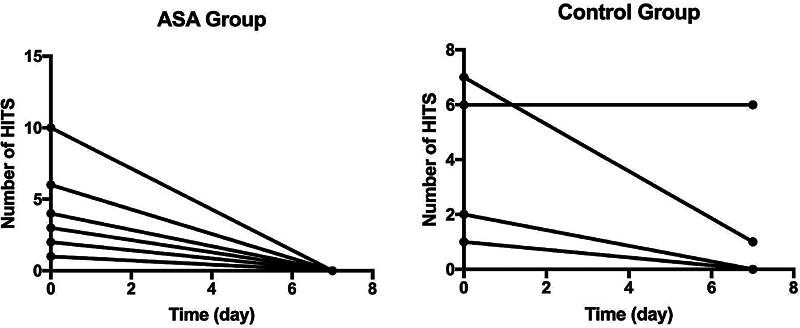
Number of high-intensity transient signals (HITS) at baseline (day 0) and 7 days in acetylsalicylic acid (ASA)-treated or control group patients.

The median number of HITS decreased from 4.0 (interquartile range [IQR] = 1.3–6.8) to 0.5 (IQR = 0.0–4.8) in the control group (within-group *p* -value = 0.095); and from 3.5 (IQR = 1.3–6.0) to none (in all patients) in the ASA-treated group (within-group *p* -value = 0.012). However, the between-group difference was not significant ( *p* = 0.262). Figure 2 shows the difference in the number of HITS between days 0 and 7.

## DISCUSSION

This proof-of-concept PROBE trial was carried out to evaluate the effectiveness of ASA in reducing the rate of cerebral microembolism in patients with Chagasic HF. The main novel finding was a significant reduction in the proportion of HITS from baseline TCD after 7 days of treatment with ASA 300 mg in comparison with the control group. Additionally, Chagas patients with HITS treated with ASA showed a significant reduction in the number of HITS, although statistical power to detect a significant difference was very low. Studies have demonstrated that antithrombotic therapy reduces the occurrence of microembolism in patients with carotid atherosclerotic disease; however, the effectiveness of ASA in reducing the rate of HITS in patients with HF, being tested as primary prophylaxis, had not been demonstrated until now. 7 8 9 14 According to the Brazilian Guidelines on Antiplatelets and Anticoagulant Agents in Cardiology, 15 warfarin is indicated for primary prevention of stroke in Chagasic patients considered at high risk, such as those with left ventricular thrombus or aneurysm. However, one cohort study actually identified the use of ASA as risk factor for stroke in patients with HF, possibly due to a deleterious effect on cardiac function. 2 Since our study only evaluated the short-term effects of ASA treatment, longer-term effects of ASA or other anti-thrombotics should be studied in Chagas disease before firm recommendations can be made.

Patients with Chagasic cardiomyopathy presented with a numerically higher proportion of HITS when compared to non-Chagasic patients, which despite agreeing with another study did not reach statistical significance. 10 Moreover, in the multivariate analysis, Chagas disease was not an independent risk factor for HITS in the first TCD. The other study 10 investigated patients with higher baseline risk of HITS, such as those with a history of stroke, which we could not include because these patients are usually already using anti-thrombotic medications. It is possible that our study was underpowered to show a difference in microembolic burden in this lower-risk population.

Other reasons for a lower-than-expected embolic risk may exist for this patient sample. Patients were younger than most stroke cohorts and predominantly female, 10 16 thus potentially lowering the microembolic burden due to the cardiovascular protective effects of endogenous estrogen in this age group. Patients with HF were predominantly in a mild/moderate functional class and frequently showed preserved left ventricular ejection fraction. Several studies have demonstrated a higher risk of embolism associated with reduced left ventricle ejection fraction, suggesting we selected a HF population with lower risk of embolism. 17 18 Chronic inflammation in Chagas disease results in endothelial dysfunction that can stimulate the hemostatic system, increasing fibrin production and platelet activation and impacting the formation of microemboli, but this was not enough to account for a statistically significant increase in HITS in our sample. 19 20 21 22

The limitations of the present study are early termination due to the repercussions resulting from the pandemic and the long duration of the study, besides underpowering due to small sample size. The open-label design was a limitation minimized through blinding of the TCD assessor, who evaluated the primary outcome of the trial. The study also suffered from some selection bias from potentially eligible patients withdrawing consent due to logistical issues in this vulnerable population (e.g., difficult access to transportation, living in a rural area). The short treatment duration of 7 days maximizes treatment efficacy (HITS reduction), so we admit long-term studies are essential to evaluate the risk/benefit of ASA. Some patients from the original cohort also suffered a stroke or died during follow-up, making them unavailable for the present trial, not being randomized and excluded from the study. Finally, monitoring by TCD was performed unilaterally in all patients using the same TCD equipment, so it is possible that bilateral or longer monitorization would have detected more HITS, increasing the study's power. However, since the same technique was used in both groups, we believe the main study results are still valid.

In conclusion, we did not replicate the finding of a higher rate of HITS in patients with Chagas disease when compared to non-Chagas patients. In the patients with Chagas disease in whom HITS were identified, seven days of ASA treatment decreased the proportion of HITS when compared to no ASA treatment, considering the pilot nature of the study, the small sample size, and the lack of a significant difference between the groups. Long-term effects of primary ASA prophylaxis in high-risk populations for stroke in Chagas disease should be further studied.
